# Data Disaggregation in Action: Filipino Americans Who Do Not Identify As Asian

**DOI:** 10.1007/s40615-025-02398-6

**Published:** 2025-04-08

**Authors:** Riti Shimkhada, Andrew Juhnke, Ninez A. Ponce

**Affiliations:** 1 Center for Health Policy Research, University of California, Los Angeles, Los Angeles, CA USA; 2 Department of Health Policy and Management, Fielding School of Public Health, University of California Los Angeles, Los Angeles, CA USA

**Keywords:** Filipino Americans, Race/Ethnicity, Data disaggregation, Asian American, Native Hawaiian and Pacific Islander, Population health surveys

## Abstract

**Background:**

The U.S. Office of Management and Budget (OMB) categorizes Filipino Americans as Asian; however, many may not identify as such, opting instead for “other” or Pacific Islander (PI). This study examines the extent to which Filipino Americans select PI or “other” rather than Asian, using a large population-based survey of Californians.

**Methods:**

We analyzed data from the 2019, 2020, and 2021 California Health Interview Survey (CHIS), the largest state health survey in the U.S. that includes write-in prompts for detailed race and ethnicity data. The focus was on participants who identified as Pacific Islanders and wrote in 'Filipino,' those who selected 'Asian' and specified 'Filipino,' and respondents who chose 'other' and wrote in 'Filipino.'

**Results:**

Our analysis included 1,859 Filipino respondents, revealing that 8.8% identified as Pacific Islander, 85.2% as Asian, and 6.1% as "other." Those identifying as PI were more likely to also identify as Latino/Hispanic, be older, and possess U.S. citizenship compared to those identifying as Asian.

**Discussion:**

Disaggregating Filipino Americans from the broader Asian category in surveys is vital for accurately identifying the community's unique needs. We recommend incorporating open-ended write-in prompts in surveys that ask respondents to first identify their broader race category (e.g. Asian). These prompts help identify and reclassify Filipino respondents who may have identified as PI. Such prompts are also important for other racial/ethnic communities who may be uncertain about how to categorize themselves. Ongoing, dynamic community-driven research is essential for understanding identities and effectively categorizing Filipino Americans and other communities.

## Introduction

Filipino Americans have long been referred to as the “forgotten Asian Americans” [1] yet they are the third largest group of Asian Americans in the U.S., with a population size of about 4.4 million [2]. Despite this large presence and their distinct community needs, Filipino Americans have received limited, albeit growing, empirical attention in population health research [3, 4]. Disaggregating Filipinos as a distinct category in health research is important as the aggregate Asian American category often masks significant disparities within the group [5]. Studies have shown that Filipinos experience higher rates of obesity, hypertension, diabetes, and other chronic conditions compared to non-Hispanic Whites and many other Asian subgroups [3, 6, 7]. Language barriers also impact healthcare access, as nearly 30% of Filipino Americans speak English less than "very well" and may require language assistance, yet they often underutilize interpreter services due to stigma or lack of awareness; further, cultural factors influence patient-provider communication, and professional medical interpreters report challenges in ensuring appropriate care for Filipino patients in the U.S. [8]. Filipinos also face occupational risks, particularly as a large segment of the U.S. healthcare workforce [9].

In 2020 Filipino Americans were thrust to newspaper headlines with reports on the toll of COVID-19 on Filipino American nurses and the larger community. The California Department of Public Health found that at the onset of the pandemic Filipino Americans accounted for at least 35% of the COVID-19 deaths among Asian American. However, they posit this estimate likely did not capture the full picture since “some Filipinos may have self-identified as Pacific Islander rather than Asian” [10]. The inclusion of Filipinos in the PI category, which tends to have small sample sizes in most population surveys, further has the potential to significantly change estimates for the PI group [11, 12].

Filipino Americans are at the perimeter between two race categories: Asian and Pacific Islanders (PI). Under the U.S. Office of Management and Budget’s (OMB) Statistical Policy Directive Number 15 (SPD 15), Filipinos are categorized as Asian [13]. Filipinos have a long history of being part of the Asian American advocacy movement in the U.S. since the 1960s [14]. But, Filipinos also share a similar colonial past with Pacific Islanders who went through similar cultural re-education that required them to speak English, change their indigenous names, and to pledge allegiance to the U.S. [15]. Given shared geography and history, it is not surprising that Filipinos might identify as PI [16]. This has important implications since accurate demographic data is crucial for policy-making, resource allocation, and addressing the needs of specific communities [14, 17, 18].

Most federal and state level surveys and data systems ask individuals to identify their race in a sequential question order wherein they are first asked to identify the aggregate race group before asking individuals to mark their disaggregated detailed race group. If a Filipino person marks PI or “Other” instead of Asian, the only way to identify that they are Filipino is if the person writes in their detailed group in an open-ended prompt. Most surveys do not have the ability to identify the degree to which Filipinos identify as PI instead of Asian because they do not offer this write-in or specify option. Open-ended questions have served as effective follow-ups to closed-ended questions and have been used in the U.S. Census survey questions on race [19].

The California Health Interview Survey (CHIS) uses the write-in option in their surveys thus allowing us to identify Filipino respondents who mark PI as their race. Using 2019–2021 CHIS data, we present here the demographics of these respondents who identify as PI as well as those who identify as Asian, and “Other”. The purpose of this study is to examine the race and ethnicity reporting behavior of Filipinos in CHIS to understand the degree to which they navigate what can be seen as an ambiguous boundary between the Asian and PI racial categories, shedding light on how racial identity is expressed in survey data and its implications for health research and policy.

## Methods

We examined data from adults in the 2019, 2020, and 2021 California Health Interview Survey (CHIS) rounds. CHIS is the largest state health survey in the United States and provides data that is representative of California’s diverse population. CHIS randomly selects one adult to interview in each randomly sampled household. The survey, conducted either on the web or by telephone, includes a comprehensive set of health-related questions.

**Fig. 1:**
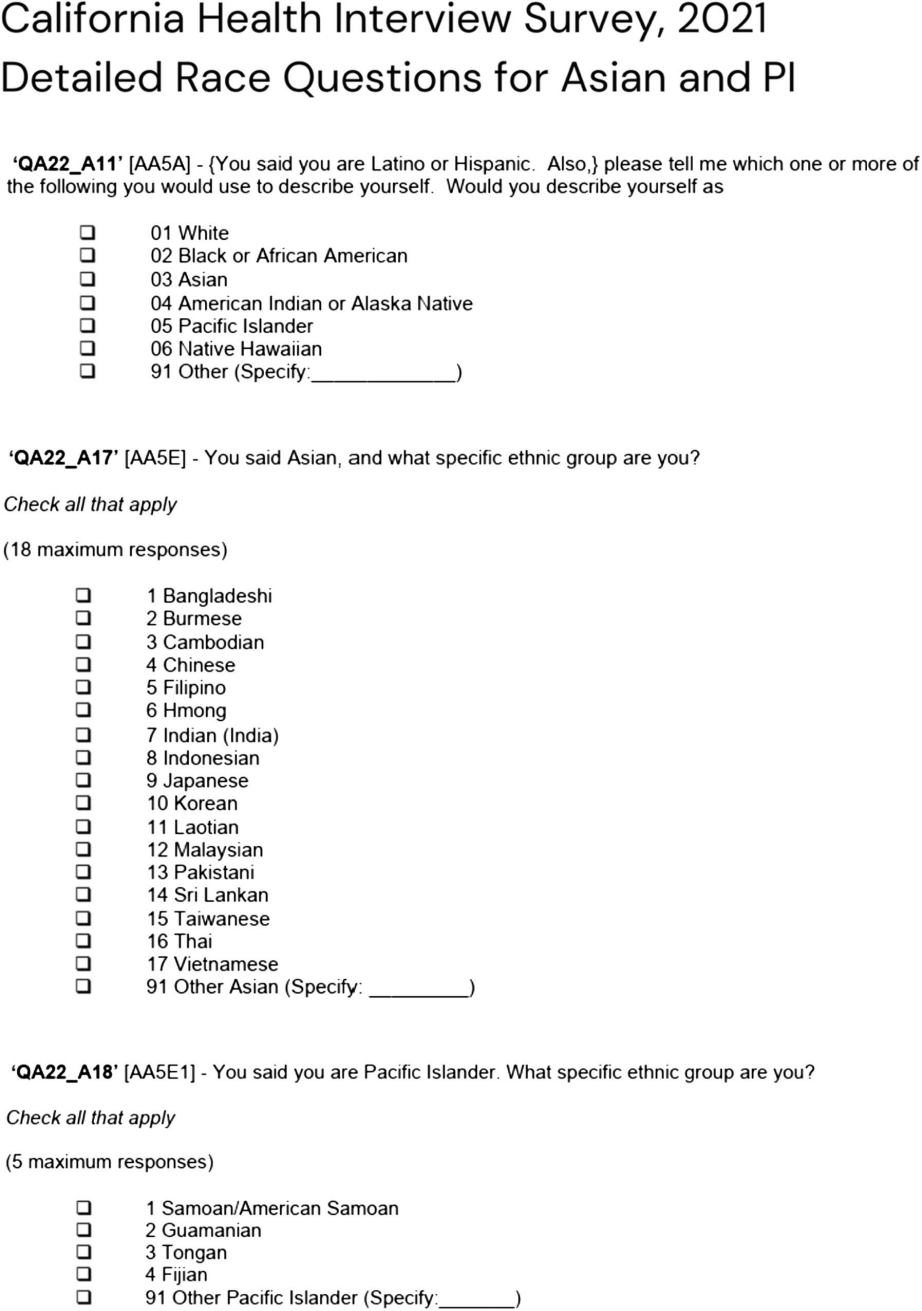
California Health Interview Survey 2021 Detailed Race Questions for Asian and Pacific Islander

CHIS collects race and/or ethnicity using a multi-part question format in line with the OMB SPD 15 guidance [14]. The data presented here reflect the OMB SPD 15 1997 guidance with data disaggregated for the Asian category [20]. From 2025 onward, CHIS data collection and reporting will align with new OMB SPD 15 guidance released in March of 2024 [13]. In the multi-part question format, the first question asks respondents if they are of Hispanic or Latino origin. If respondents answer yes, they are asked to select one or more Hispanic or Latino subgroups from a list. The second question asks respondents to select one or more race categories: American Indian or Alaska Native, Asian, Black or African American, Native Hawaiian, Pacific Islander, and White. Respondents can select one or more of these categories and can select an option for "other" race, which allows them to write-in their specific race if it is not on in the list. For respondents who select Asian or Pacific Islander categories, they are subsequently asked to select more detailed subgroup. For Asians, they can select Asian Indian, Chinese, Filipino, Japanese, Korean, Vietnamese, or “Other Asian” with a write-in prompt, among others. For Pacific Islanders, they can select Samoan/American Samoan, Guamanian, Tongan, Fijian, or “Other Pacific Islander” with a write-in prompt (see Fig. 1).

We were interested here in those respondents who selected the category Pacific Islander and specified Filipino in the write-in. We also examined the respondents who selected the aggregate Asian category as their race and chose Filipino as their detailed race group and respondents who selected the “other” category as their aggregated race and wrote-in Filipino. We calculated descriptive statistics for these groups using CHIS variables on Latino/Hispanic ethnicity, gender, age, poverty (based on the Federal Poverty Level, which is constructed using reported household income adjusted to household size), and U.S. citizenship. Data presented here are unweighted. As this analysis is descriptive in nature, hypothesis test statistics were not conducted.

We also examined large on-going federal and state data systems to determine which ones include a write-in or specify prompt for any of their “other” categories for questions pertaining to race and ethnicity. These surveys included: Behavioral Risk Factor Surveillance System (BRFSS), Consumer Assessment of Healthcare Providers and Systems (CAHPS), Medical Expenditure Panel Survey (MEPS), Medicare Current Beneficiary Survey (MCBS), National Health and Nutrition Examination Survey (NHANES), National Health Interview Survey (NHIS), National Immunization Survey (NIS), National Survey of Children’s Health (NSCH), National Survey of Family Growth Survey (NSFG), National Survey on Drug Use and Health (NSDUH), National Youth Tobacco Survey (NYTS), National Vital Statistics System (NVSS), and Youth Risk Behavior Surveillance System (YRBSS).

## Results

Our sample consisted of 1,859 respondents in 2019–2021 CHIS who identified as Filipino (Table 1). Of this group, 8.8% (*n *= 163) identified as Pacific Islander, 85.2% (*n *= 1,583) identified as Asian and 6.1% (*n *= 113) identified as “other” race for their larger/aggregate group racial identity.

**Table 1:** Filipino respondents in 2019–2021 CHIS, n=1,859, unweighted results on the sample of respondents

		Filipinos identifying as:		
	All Filipinos (*n* = 1859)	Pacific Islander (*n *= 163)	Other (*n *= 113)	Asian (*n *= 1,583)
Latino or Hispanic	6.4%	16.6	17.7	4.5
Gender				
Male	41.3	47.2	29.2	41.5
Female	58.7	52.8	70.8	58.5
Age				
18–29	11.5	3.2	3.6	12.6
30–49	34.7	37.6	28.2	35.1
50–69	39.5	40.1	54.6	38.6
70+	14.3	19.1	13.6	13.6
Federal Poverty Line (FPL)				
0–99%	7.0	7.4	9.7	6.8
100–199%	11.6	11.7	11.5	11.6
200–299%	12.0	11.0	11.5	12.1
300% +	69.4	69.9	67.3	69.5
U.S. Citizenship				
Citizen, U.S. born	34.3	54.6	53.1	30.9
Citizen, Naturalized	57.3	42.3	46.0	59.7
Non-citizen with green card	6.7	n/a	n/a	7.6
Non-citizen without green card	1.6	n/a	n/a	1.8

n/a is not available due to small sample size

About 16.6% of Filipinos identifying as Pacific Islander reported Latino or Hispanic identity, compared to about 4.5% among Filipinos who identified as Asian. There was a higher percent of older age Filipinos over 70 years identifying as Pacific Islander (19.1%) compared to those identifying as Asian (14.3%). Lastly, Filipinos identifying as Pacific Islander tended to be U.S. born citizens (54.6%) compared to those identifying as Asian (30.9%).

In our examination of thirteen large federal and state health surveys we found only the National Survey on Drug Use and Health (NSDUH) and the National Vital Statistics System (NVSS) offer respondents opportunities to write-in or specify their identities in an open-ended “other” prompt.

## Discussion

Our work is the first to present data on the degree to which Filipinos identify as PI from a population-based survey setting. We found about 9% of Filipinos identified as PI. Our findings are consistent with an earlier study conducted in a clinical setting in Hawaii where about 11% of single-race Filipinos did not identify as Asian, and instead, marked Native Hawaiian or Pacific Islander (NHPI) [16].

We found about 85% of Filipinos identified as Asian, and about 6% of Filipinos identified as neither Pacific Islander or Asian opting to self-identify as “other” as their large group racial identity. These findings are consistent with prior research that suggest Filipinos, as well as other Southeast Asians, have often felt misclassified being placed under the larger Asian American designation. Non-Asians tend view Filipinos as not Asians [21]. Further work has shown that East Asians may also have the tendency to exclude Filipinos from the Asian American category [22].

Given the unique situation of Filipino Americans and likely other smaller racial/ethnic groups facing similar challenges in determining which categories best describe them, we recommend (1) the wider use of write-in prompts for questions pertaining to race and ethnicity where a respondent can fill in what “other” racial or ethnic group they identify with. These open-ended prompts may function as primary inquiries in situations where closed-ended questions may fall short in extracting necessary information [23]. In the OMB SPD 15’s 2024 updated guidance, the Federal Interagency Technical Working Group on Race and Ethnicity Standards recommends detailed collection of race/ethnicity, which includes disaggregated data categories and write-in fields [13]. They stress the need to adopt practices that enhance comparability between race/ethnicity data collected with and without write-in fields, improving consistency across datasets. These practices should include standards on how data producers identify and rectify potentially misclassified respondents per OMB 1997 race and ethnicity data collection guidance. As part of CHIS’ annual data cleaning and processing, Filipinos who have written-in their identities under the “other” categories are found and then brought into the Filipino subgrouping. Bringing these respondents into the appropriate grouping is important given the large number of respondents that would have been missing completely from this category if they hadn’t been identified through this process, contributing to statistically robust and accurate statistics for the group. NVSS state systems similarly have data quality guidelines or algorithms to identify groups based on the write-in responses to group them into the appropriate categories, which may be made accessible to the public [24]. The U.S. Census Bureau published technical documentation of their race/ethnicity code list from write-in responses in the 2020 U.S. Census, which has enhanced transparency in its methods for categorizing different racial and ethnic groups [25]. Further work in establishing guidance for write-in responses could start with a compilation of such best practices.

We further recommend that: (2) Filipino Americans should always be disaggregated from the Asian category. Detailed ethnic subgroups listed for Asians should be drawn from the newly updated OMB SPD 15 guidelines as the minimum standard for detailed data collection for the Asian category, which includes Asian Indian, Chinese, Filipino, Japanese, Korean, Vietnamese, and Another group. The need for this level of disaggregation has been supported by a large body of literature showing the high degree of diversity in the Asian category is and how unique needs of smaller subgroups are obscured in the aggregate form [5, 26].

When sample sizes are too small to report the smallest disaggregated categories, public-facing may be best presented in intermediate categories rather than the largest broad category. This approach allows for a more nuanced understanding of the diverse Asian populations while maintaining compliance with OMB standards. By grouping individuals into broader regional categories—such as East Asian, Southeast Asian, and South Asian—data can be presented in a way that reflects the unique characteristics and needs of these communities.

This method not only enhances the granularity of the data but also ensures that smaller populations are not overlooked or rendered invisible due to insufficient sample sizes. Furthermore, these intermediate categories can be rolled up into the broader Asian category when necessary, allowing for consistency with OMB standards while still providing valuable insights into the specific demographics and experiences of various Asian subgroups. Implementing this strategy can improve data collection and reporting practices, ultimately leading to better-informed policies and programs that address the distinct needs of different Asian communities. For example, intermediate categories might include: Central Asian, East Asian, Southeast Asian, and South Asian. In such a categorization, the Filipino subgroup could be part of the Southeast Asian category along with Burmese, Cambodian, Hmong, Indonesian, Laotian, Malaysian, Thai, and Vietnamese. Further disaggregation may be warranted within these intermediate categories, for example distinguishing refugee versus non refugee populations, and populations with colonial versus non-colonial histories [27].

We have identified several studies from Hawai’i where Native Hawaiian, Pacific Islander (NHPI), and/or Filipino populations are grouped together to create a category often referred to as NHPIF [28–33]. In some instances, this grouping is referred to as Indigenous Pacific People (IPP) [34]. These studies often state NHPI and Filipinos share status as underrepresented and understudied groups in Hawaii, as such this grouping might be suitable for the research questions examined in these studies and the Hawaiian context in particular. This grouping may not be suitable for other questions or settings, however, thus careful consideration should be made in making decisions regarding aggregation. For example, grouping NHPI with Filipino might mask the high mental health needs of NHPI communities in California [35]. Clear documentation and thoughtful categorization of racial and ethnic identities are crucial for maintaining research accuracy and integrity, as they help prevent the misclassification or exclusion of groups that may mask health disparities. Yi (2024) emphasizes that researchers should transparently disclose their classification decisions and provide justification for their methodological choices to promote equitable and meaningful analysis [36]. How communities are grouped significantly impacts the results and interpretations of research and we have ample research to show that aggregating data without careful consideration can obscure important differences and lead to misleading conclusions [5–7, 18, 26, 37–40].

We recommend further work in this area by working closely with community groups ensure communities feel represented or included in the recommended groupings. The OMB SPD 15 update in 2024 explicitly calls for research and stakeholder engagement in creating intermediate categories [13]. And, notably a Federal Register notice by the U.S. Census Bureau in 2024 sought public comment regarding its proposed race and ethnicity code list with a specific solicitation for input on regional groupings [41]. The impetus of this call can be traced to the 2020 Census race/ethnicity code list that organized groups into regional categories, such as aggregating Chinese and Japanese individuals into East Asian, with counts for these categories published in the Census data products. However, based on stakeholder feedback regarding the complexities and ambiguities of defining regional boundaries – with particular concern from the Hmong community as they were placed in the East Asian grouping rather than Southeast Asian - the Census Bureau proposed to eliminate these predefined regional categories. This change aims to provide data users with greater flexibility to create their own regional classifications using disaggregated data, which could enhance the relevance and applicability of the data for various research and policy purposes. Our work serves as a call for further studies on how best to create such regional categories, including considerations of historical context for migration to the US and community-driven discussion on how to tabulate data for communities that may be obscured by prevailing methods. The implications of this process extend beyond mere categorization as they influence the broader discourse on race and ethnicity in American society.

## Data Availability

The data used in this study are from the California Health Interview Survey (CHIS), which is publicly available (https://healthpolicy.ucla.edu/chis). Analyses presented in this study of detailed race and ethnicity required access through CHIS’s Data Access Center.

## References

[1] Cordova F. Filipinos: Forgotten Asian Americans. Dubuque, IA: Kendall/Hunt. 1983.

[2] U.S. Census Bureau. Asian alone or in any combination by selected groups. 2021 ACS 1-year estimates detailed tables. US Census Bureau. 2021. Available at: https://data.census.gov/. Accessed on 12 Sept 2024

[3] Adia AC, Restar AJ, Nazareno J, et al. Asian, Latinx, or Multiracial? Assessing Filipinxs’ health conditions and outcomes by aggregate ethnic category. J Racial Ethn Health Disparities. 2022;9(2):406–12. 10.1007/s40615-021-00971-3. 33594653 10.1007/s40615-021-00971-3

[4] Sabado-Liwag MD, Manalo-Pedro E, Taggueg R Jr, et al. Addressing the interlocking impact of colonialism and racism on Filipinx/a/o American health inequities. Health Aff (Millwood). 2022;41(2):289–95. 10.1377/hlthaff.2021.01418. 35130069 10.1377/hlthaff.2021.01418

[5] Ðoàn LN, Chau MM, Ahmed N, Cao J, Chan SWC, Yi SS. Turning the health equity lens to diversity in Asian American Health Profiles. Annu Rev Public Health. 2024;45(1):169–93. 10.1146/annurev-publhealth-060222-023852. 38134402 10.1146/annurev-publhealth-060222-023852

[6] Adia AC, Nazareno J, Operario D, Ponce NA. Health conditions, outcomes, and service access among Filipino, Vietnamese, Chinese, Japanese, and Korean Adults in California, 2011–2017. Am J Public Health. 2020;110(4):520–6. 10.2105/ajph.2019.305523. 32078359 10.2105/AJPH.2019.305523 PMC7067106

[7] Jiang JJ, Adia AC, Nazareno J, Operario D, Ponce NA, Shireman TI. The association between moderate and serious mental health distress and general health services utilization among Chinese Filipino, Japanese, Korean, and Vietnamese adults in California. J Racial Ethn Health Disparities. 2022;9(1):227–35. 10.1007/s40615-020-00946-w. 33452574 10.1007/s40615-020-00946-w

[8] Kostareva U, Soo Hoo CA, Zeng SM, Albright CL, Ceria-Ulep CD, Fontenot HB. Understanding professional medical interpreters' perspectives on advancing accurate and culturally informed patient-provider communication for Filipinos in Hawai'i: qualitative analysis. Int J Environ Res Public Health. 2023; 20(21), 10.3390/ijerph20217012. 10.3390/ijerph20217012 PMC10649552 37947568

[9] Oronce CIA, Adia AC, Ponce NA. US Health care relies on Filipinxs while ignoring their health needs: disguised disparities and the COVID-19 Pandemic. JAMA Health Forum. 2021;2(7):e211489. 10.1001/jamahealthforum.2021.1489. 36218776 10.1001/jamahealthforum.2021.1489

[10] California Department of Public Health. Comment letter on proposed rule for updating race and ethnicity statistical standards posted by the office of management and budget (OMB-2023-0001-0001), accessed April 2023. Available at: https://www.regulations.gov/comment/OMB-2023-0001-16960. Accessed on 12 Sept 2024

[11] UCLA Center For Health Policy Research Native Hawaiian and Pacific Islander Data Policy Lab. Comment letter on proposed rule for updating race and ethnicity statistical standards posted by the office of management and budget (OMB-2023-0001-0001), UCLA Center For Health Policy Research Native Hawaiian and Pacific Islander Data Policy Lab. 2023. Available at: https://www.regulations.gov/comment/OMB-2023-0001-16983. Accessed on 12 Sept 2024

[12] Data Equity Center (DEC) UCLA Center For Health Policy Research. Comment Letter on proposed rule for updating race and ethnicity statistical standards posted by the office of management and budget (OMB-2023-0001-0001). Data Equity Center (DEC) UCLA Center For Health Policy Research. 2023. Available at: https://www.regulations.gov/comment/OMB-2023-0001-16984. Accessed on 12 Sept 2024

[13] US Office of Management and Budget. Revisions to OMB's statistical policy directive no. 15: standards for maintaining, collecting, and presenting federal data on race and ethnicity. 2024. https://www.federalregister.gov/documents/2024/03/29/2024-06469/revisions-to-ombs-statistical-policy-directive-no-15-standards-for-maintaining-collecting-and,US-Office-of-Management-and-Budget. Accessed on 12 Sept 2024

[14] Ponce NA. Centering health equity in population health surveys. JAMA Health Forum. 2020; 1(12):e201429. 10.1001/jamahealthforum.2020.1429. 10.1001/jamahealthforum.2020.1429 36218466

[15] Strobel LM. Coming full circle: the process of decolonization among post-1965 Filipino-Americans. Manila: Giraffe Books; 2001.

[16] Holup JL, Press N, Vollmer WM, Harris EL, Vogt TM, Chen C. Performance of the U.S. Office of Management and Budget’s Revised Race and Ethnicity Categories in Asian Populations. Int J Intercult Relat. 2007; 31(5):561-573. 10.1016/j.ijintrel.2007.02.001. 10.1016/j.ijintrel.2007.02.001 PMC2084211 18037976

[17] Mays VM, Ponce NA, Washington DL, Cochran SD. Classification of race and ethnicity: implications for public health. Annu Rev Public Health. 2003;24:83–110. 10.1146/annurev.publhealth.24.100901.140927. 12668755 10.1146/annurev.publhealth.24.100901.140927 PMC3681827

[18] Ponce NA, Shimkhada R, Adkins-Jackson PB. Making communities more visible: equity-centered data to achieve health equity. Milbank Q. 2023;101(S1):302–32. 10.1111/1468-0009.12605. 37096622 10.1111/1468-0009.12605 PMC10126976

[19] Institute of Medicine Subcommittee on Standardized Collection of Race/Ethnicity Data for Healthcare Quality Improvement, Ed. Race, Ethnicity, and Language Data: Standardization for Health Care Quality Improvement. Defining categorization needs for race and ethnicity data. Washington (DC): National Academies Press (US); 2009. Available from: https://www.ncbi.nlm.nih.gov/books/NBK219754/. Accessed on 12 Sept 2024

[20] US Office of Management and Budget. Revisions to the standards for the classification of federal data on race and ethnicity. US Office of Management and Budget. 1997. Available at: https://www.govinfo.gov/content/pkg/FR-1997-10-30/pdf/97-28653.pdf. Accessed on 12 Sept 2024

[21] Lee J, Ramakrishnan K. Who counts as Asian. Ethnic and Racial Studies. 2020;43(10):1733–56. 10.1080/01419870.2019.1671600.

[22] Ocampo AC. The Latinos of Asia: how Filipino Americans break the rules of race. Stanford University Press. 2020.

[23] Zipp L. Questionnaires to elicit qualitative data. In: Kircher R, Zipp L, editors. Research methods in language attitudes. Cambridge: Cambridge University Press; 2022. p. 145–59.

[24] NYC Health, Office of Vital Statistics. Ancestry and Race Data Items in the Electronic Birth Registration System (EBRS). 2010. https://www.nyc.gov/assets/doh/downloads/pdf/vr/vr-preg-his-newsletter.pdf. Accessed on 12 Sept 2024

[25] US Census Bureau. 2020 Census National Redistricting Data Summary File. Technical Documentation. 2020 Census of Population and Housing. SFNRD/20-02. 2021.

[26] Shimkhada R, Scheitler AJ, Ponce NA. Capturing racial/ethnic diversity in population-based surveys: data disaggregation of health data for Asian American, Native Hawaiian, and Pacific Islanders (AANHPIs). Popul Res Policy Rev. 2021;40(1):81–102. 10.1007/s11113-020-09634-3.

[27] Gee GC, Chien J, Sharif MZ, Penaia C, Tran E. East is east … or is it? Racialization of Asian, Middle Eastern, and Pacific Islander persons. Epidemiol Rev. 2023;45(1):93–104. 10.1093/epirev/mxad007. 37312559 10.1093/epirev/mxad007 PMC13376132

[28] Fialkowski MK, Ng-Osorio J, Kai J, et al. Type, timing, and diversity of complementary foods among native Hawaiian, Pacific Islander, and Filipino infants. Hawaii J Health Soc Welf. 2020;79(5 Suppl 1):127–34. 32490400 PMC7260875

[29] Johnson DL, Okamoto SK, Rosario MH, Pokhrel P. Tobacco product use and cultural connectedness among Native Hawaiian/Pacific Islander, Asian American, and Filipino American young adults in Hawai’I. J Ethn Subst Abuse. 2024;23(4):967–81. 10.1080/15332640.2022.2161082. 36579697 10.1080/15332640.2022.2161082 PMC10307923

[30] Kai J, Chen JJ, Braun KL et al. Associations between cultural identity, household membership and diet quality among native Hawaiian, Pacific Islander, and Filipino infants in Hawai'i, Children (Basel). 2022; 9(1). 10.3390/children9010048. 10.3390/children9010048 PMC8774442 35053673

[31] Mulville K, Kai J, Kearney JM, Ng-Osorio J, Boushey CJ, Fialkowski MK. A qualitative analysis of a caregivers' experience of complementary feeding in a population of native Hawaiian, other pacific islander and filipino infants: the timing of the introduction of complementary foods, and the role of transgenerational experience. Nutrients. 2022; 14(16). 10.3390/nu14163268. 10.3390/nu14163268 PMC9412982 36014772

[32] Shimokawa MAL, Siu AM, Choi SY, Davis J. The NEW Keiki Program reduces BMI z-scores among overweight and obese children and BMI among their adult caregivers in Hawai’i. Hawaii J Health Soc Welf. 2020;79(5 Suppl 1):24–31. 32490382 PMC7260860

[33] Vakalahi HO, Okamoto SK, Horgen FD, et al. Promoting health research among underrepresented students through the HUI SRC. Hawaii J Health Soc Welf. 2023;82(10 Suppl 1):36–43. 37901664 PMC10612418

[34] Glauberman G, Mendoza Kabua P, Camba M, Dela Cruz M, Fontenot HB. Perspectives on emergency preparedness among indigenous Pacific people in Hawaii: a qualitative study. J Community Health Nurs. 2024;41(3):189–202. 10.1080/07370016.2024.2309375. 38334130 10.1080/07370016.2024.2309375 PMC11128344

[35] Tan C, Lo F, Ponce NA, Ocampo C, Galan M. Piecing the Puzzle of AANHPI mental health: a community analysis of mental health experiences of Asian Americans, Native Hawaiians, and Pacific Islanders in California. 2024. https://healthpolicy.ucla.edu/sites/default/files/2024-02/final-chis-report-designed-2.26.24.pdf. Accessed on 12 Sept 2024

[36] Yi SS. data equity and multiracial and multiethnic communities. JAMA Netw Open. 2024;7(11):e2446839. 10.1001/jamanetworkopen.2024.46839. 39576649 10.1001/jamanetworkopen.2024.46839

[37] Lui CK, Ye Y, Gee J, et al. Unmasking suicidal ideation for Asian American, Native Hawaiian, and Pacific Islander youths via data disaggregation. JAMA Netw Open. 2024;7(11):e2446832. 10.1001/jamanetworkopen.2024.46832. 39576641 10.1001/jamanetworkopen.2024.46832 PMC11584931

[38] Ponce NA, Becker T, Shimkhada R. Breaking barriers with data equity: the essential role of data disaggregation in achieving health equity. Annu Rev Public Health. 2025. 10.1146/annurev-publhealth-072523-093838. Accessed on 12 Sept 2024 10.1146/annurev-publhealth-072523-093838 39883940

[39] Kauh TJ, Read JG, Scheitler AJ. The critical role of racial/ethnic data disaggregation for health equity. Popul Res Policy Rev. 2021;40(1):1–7. 10.1007/s11113-020-09631-6. 33437108 10.1007/s11113-020-09631-6 PMC7791160

[40] Kauh TJ, Minnis TA, Anand M, Berry M, Gold R. Building an equitable future through data disaggregation. Health Equity. 2023;7(1):251–60. 10.1089/heq.2023.29036.rtd. 37096058 10.1089/heq.2023.29036.rtd PMC10122218

[41] US Census Bureau. The Census Bureau’s proposed race/ethnicity code list for the american community survey and the 2030 Census. 2024. https://www.federalregister.gov/documents/2024/11/18/2024-26827/the-census-bureaus-proposed-raceethnicity-code-list-for-the-american-community-survey-and-the-2030. Accessed on 12 Sept 2024

